# Novel Superdielectric Materials: Aqueous Salt Solution Saturated Fabric

**DOI:** 10.3390/ma9110918

**Published:** 2016-11-11

**Authors:** Jonathan Phillips

**Affiliations:** Energy Academic Group, Naval Postgraduate School, Monterey, CA 93943, USA; jphillip@nps.edu

**Keywords:** dielectric, capacitance, energy storage

## Abstract

The dielectric constants of nylon fabrics saturated with aqueous NaCl solutions, Fabric-Superdielectric Materials (F-SDM), were measured to be >10^5^ even at the shortest discharge times (>0.001 s) for which reliable data could be obtained using the constant current method, thus demonstrating the existence of a third class of SDM. Hence, the present results support the general theoretical SDM hypothesis, which is also supported by earlier experimental work with powder and anodized foil matrices: Any material composed of liquid containing dissolved, mobile ions, confined in an electrically insulating matrix, will have a very high dielectric constant. Five capacitors, each composed of a different number of layers of salt solution saturated nylon fabric, were studied, using a galvanostat operated in constant current mode. Capacitance, dielectric constant, energy density and power density as a function of discharge time, for discharge times from ~100 s to nearly 0.001 s were recorded. The roll-off rate of the first three parameters was found to be nearly identical for all five capacitors tested. The power density increased in all cases with decreasing discharge time, but again the observed frequency response was nearly identical for all five capacitors. Operational limitations found for F-SDM are the same as those for other aqueous solution SDM, particularly a low maximum operating voltage (~2.3 V), and dielectric “constants” that are a function of voltage, decreasing for voltages higher than ~0.8 V. Extrapolations of the present data set suggest F-SDM could be the key to inexpensive, high energy density (>75 J/cm^3^) capacitors.

## 1. Introduction

Two parallel, but technically distinct, efforts are underway for improving the energy density of capacitors: (i) employing electrically conductive materials with the highest possible surface areas as electrodes (EDLC, also known as supercapacitors); and (ii) finding/inventing electrically insulating materials with higher dielectric constants. In contrast to EDLC, the electrodes area in the high dielectric constant capacitors are quite low, approximately equal the macroscopic surface area. Increasing capacitance via the former route may have reached a limit with the deployment of graphene, “single layer” electrically conductive graphitic carbon, with a theoretical surface area limit of ~2600 m^2^/gm. Until recently, efforts via the latter route focused primarily on improving barium titanate. Arguably this approach was not successful and significant improvements in energy storage density were not achieved. However, the recent invention of super dielectric materials, which employ dispersed liquids containing dissolved salts, not solids, as the dielectric (SDM) has dramatically improved energy density in capacitors based on this second approach. Indeed, the published energy density of prototypes of the two approaches are similar, with graphene based EDLC approaching 450 J/cm^3^, and SDM based Novel Paradigm Supercapacitors (NPS) using aqueous solutions of NaCl in a anodized titania matrix as the dielectric with energy density of nearly 400 J/cm^3^ [1,2].

Previously, two types of materials were demonstrated to have dielectric constants greater than 10^5^, and hence qualify as super dielectric materials (SDM): (i) porous oxides (e.g., alumina) filled with aqueous salt solutions [3,4,5], so called Powder SDM (P-SDM); and (ii) anodized titania films, “Tube” SDM (T-SDM), also filled with aqueous salt solutions [1,2]. The measured dielectric constants of materials described in those studies was >10^10^ in many cases, hence orders of magnitude higher than required to meet the required minimum, greater than 10^5^, for a material to be classified as an SDM. In the present study a prediction of the general SDM hypothesis was tested: Any material composed of liquid containing dissolved, mobile ions, confined in an electrically insulating matrix, will have a very high dielectric constant. Specifically, a novel type of material that fits the above description was studied as a dielectric; nylon fabric saturated with aqueous NaCl salt solutions. Five capacitors composed of layers of this material were studied and each performed as superdielectric over the entire range of discharge rates studied. Once again, for slow discharge (ca. 100 s), dielectric constants as high as 10^11^ were measured, and, even at the shortest discharge rates, 0.001 s, no dielectric constant <10^7^ was measured. Thus, the present work supports the general SDM hypothesis.

Potentially, F-SDM are attractive engineering materials: inexpensive (fabric and salt water), easy to fabricate, and with a potential for high energy density. However; in order to better assess the applicability of F-SDM for fast discharge processes, the “roll-off” of capacitance with discharge time was studied.

The very theory of SDM suggests they may be relatively slow to release energy. SDM are based on diffusion of ions inside the liquid filling the pores of the electrically insulating matrix material to form “giant” (ca. microns in length) dipoles upon exposure to an electric field. It takes time for the ions to move and form the multi-micron length dipoles required to explain super dielectric behavior. That is, ions initially randomly dispersed in a liquid must travel the order of the thickness of the dielectric layer (many microns in all cases studied to date) to ultimately create giant dipoles. Similarly, once formed, it takes time for the dipoles to “dissolve” allowing electrons on the electrodes to release. This model of multi-micron ionic travel under the influence of an applied field suggests there should be a time dependence to the capacitance of SDM. Qualitatively, it is anticipated that if the applied field is switched too quickly from one polarity to the opposite, the dipoles will not achieve their maximum possible magnitude. That is, the ions that form the dipole will simply not have time to travel to their equilibrium, fully polarized, positions. This in turn implies smaller effective dipoles at higher frequency, hence a lower effective dielectric constant. This should lead to a smaller measured capacitance with increasing frequency. One major objective of the present work is to quantify the effect of frequency on capacitance.

The present data, based on constant current analysis between 2.3 and 0.1 volts, clearly show there is a strong roll-off of capacitance for F-SDM. The capacitance, from ~1 volt, at ~10 Hz is of the order of 1 percent that at ~0.01 Hz or to put it another way, the capacitance for a discharge time of 100 s is about 100 times greater than the capacitance for a discharge of 0.1 s. The dielectric constant shows nearly an identical rate of roll off, and the energy density measured over the full voltage range shows a slightly higher rate of roll off. This suggests the forming process for full strength dipoles in F-SDM take of the order of tens of seconds. This type of information clearly will help determine the suitability of F-SDM for particular applications.

## 2. Results

**Figure 1:**
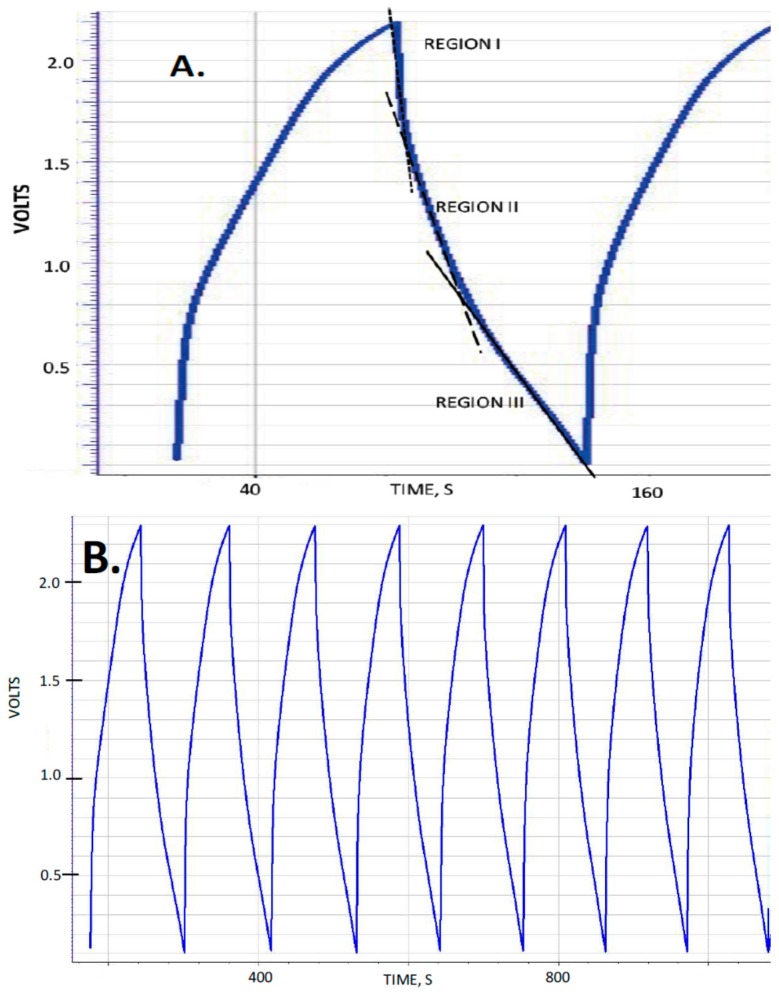

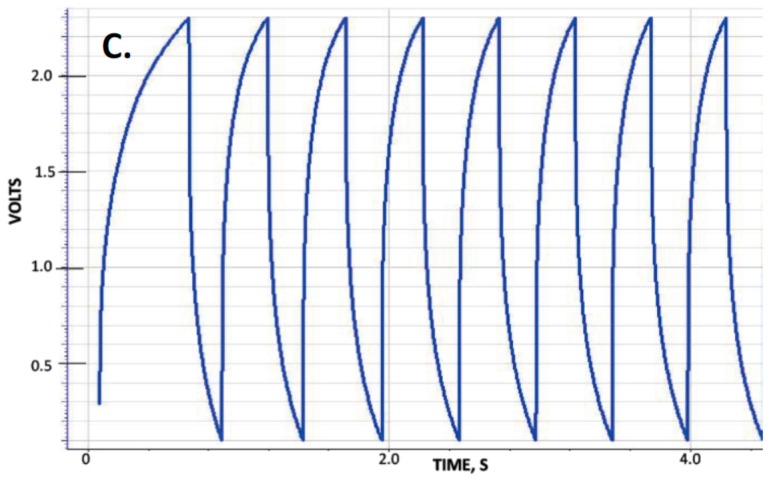
Five Layers of Nylon Cycles: (**A**) 12 mA, DT ~ 54 s showing one cycle, and illustrating three voltage regions; (**B**) 12 mA, DT ~ 54 s, 7 cycles; and (**C**) 90 mA, DT ~ 0.25 s, 7 cycles. As shown, the first ~3 cycles collected at any current value are “not typical” and not used in computation. Note: Data were collected on the basis of the smaller of these two values, 0.01 volts or 0.01 s, in all cases. (The plotting routine for this figure lumped data to approximately one value every 0.5 s.)

The capacitance, measured dielectric constant, energy density and power delivery characteristics of five capacitors were studied as a function of discharge time (DT) defined as the time the voltage takes, during the discharge half-cycle, to drop from 2.3 to 0.1 volts. These capacitors were designed to be nearly identical in all respects except for the thickness, corresponding to the number of layers of nylon employed to create the dielectric layer (Table 1). Typical observed behavior for the “constant current” tests is shown in Figure 1 for several DT. A material with a voltage independent dielectric constant will produce a straight line, and it was consistently found that for the F-SDM studied herein, that is only approximately the case for DT > 50 s. As with previous studies of SDM materials, the dielectric constant is found to vary with voltage for DT less than about 50 s. Indeed, there are roughly three regions of near constant dielectric constant: 2.3–1.6 V (Region I), 1.6–0.8 volts (Region II) and <0.8 volts (Region III). For the present study, the dielectric constant values presented are those of Region III.

**Figure 2:**
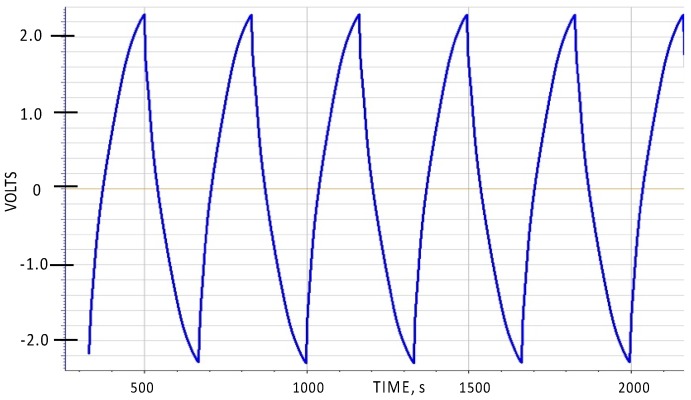
Symmetric Capacitance. NPS capacitors are symmetric, hence can be charged and discharged from −2.3 to +2.3 Volts. Data shown are from a five-layer sample, operated at 12 mA constant current mode.

One general aspect of SDM behavior not described in earlier papers is the symmetry of charge/discharge behavior with respect to the polarity of the voltage. As Novel Paradigm Supercapacitors (NPS), that is capacitors created from a sandwich of two low surface area electrodes with an SDM dielectric between, are completely symmetric, the expectation is that the charge/discharge behavior will be symmetric with respect to the sign of the voltage. This is shown to be true in Figure 2. As shown, the voltage limits were set to be from −2.3 to +2.3 volts, whereas in all prior reports of SDM behavior the voltage was never permitted to drop below zero volts.

### 2.1. Capacitance

**Figure 3:**
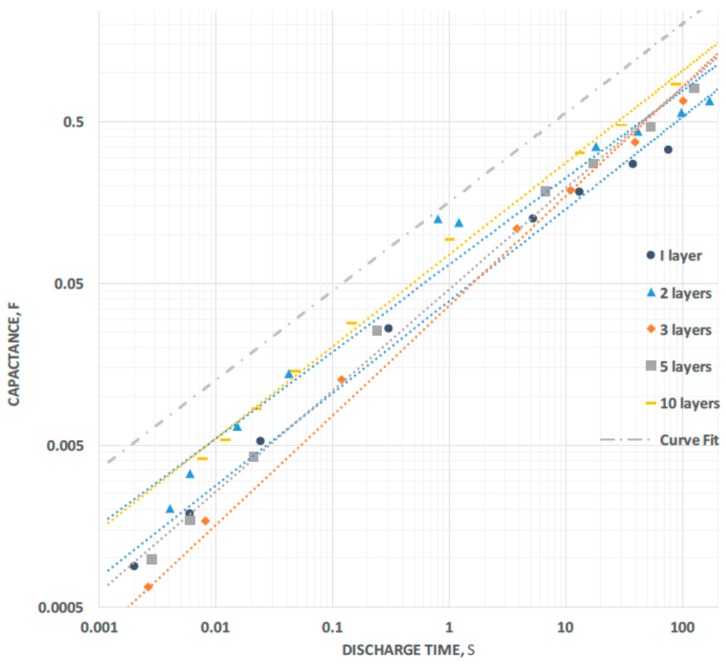
Capacitance vs. Discharge Time. For long discharge time (>100 s) capacitance increases with each additional layer, but for all capacitors, except three-layer, approximately the same simple power relationship (slope) between discharge time and capacitance describes the capacitive roll-off with decreasing discharge time. The dashed curve (above others) is the fit to Equation (1).

Capacitance for the voltage Region III is shown in Figure 3 for all five capacitors tested. In all cases, the capacitance “rolls off” very smoothly as a power law with decreasing discharge time. Except for the three-layer case, the same power law describes the roll off of all the test capacitors very well:

Capacitance = C_100_ × (100/DT)^−0.55^ (1)

where C_100_ is the capacitance at 100 s, and DT is the discharge time. Roughly, this is equivalent to a roll off of 0.55 dB capacitance for 1.0 dB decrease in “period”. The “curve fit” line of Equation (1) is the dashed curve in Figure 3. The same relationship also fits the dielectric data, substituting dielectric constant for capacitance and “D_100_” for C_100_. In addition, nearly the same relationship fits virtually all the energy data (“E_100_” for C_100_), except the exponent is changed from −0.55 to −0.60, indicating a slightly faster energy roll off.

### 2.2. Dielectric Constant

**Figure 4:**
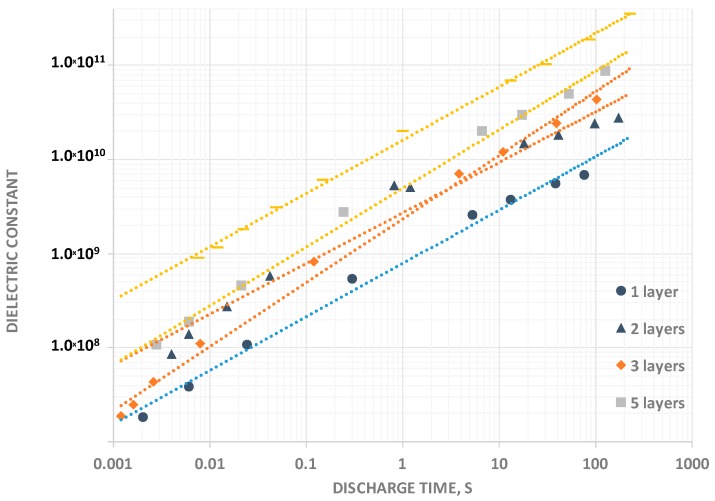
Dielectric Constant vs. Discharge Time. In all cases, even for discharge times of order 0.001 s, the measured dielectric constant was >10^7^. At relatively long discharge times, >100 s, the dielectric constants were, remarkably, >10^10^ in all cases.

The variation of the dielectric constant in Region III is an important parameter, but does not tell the entire story (Figure 4). The dielectric constant decreases with increasing voltage, and the ratio of the dielectric constants in the regions is a function of DT. For a DT of approximately 1 s, the dielectric constant for the Region III is twice that for Region II and four times that for Region I. For a DT of 0.01 s, the dielectric constant for Region III is three time that for Region II and 10 times that of Region I.

There is a clear trend: as the DT gets shorter, the voltage drops more quickly in Regions I and II relative to Region III. More detail on this behavior is found in earlier articles [1,2,3,4,5]. Rather than focus on this complex behavior, in this paper, the trends in total energy density, and power as a function of DT, which includes contributions from all voltage regions, is reported. The total energy and power are “lumped” data from all voltage regions. This lumping is necessary to more readily describe “non-textbook”, that is voltage dependent capacitance, behavior. Moreover, the lumped values reflect net energy and power behavior as a function of the discharge rate, and concomitantly frequency. The lumped values are significant indicators of performance in the likely applications: energy storage and/or power delivery.

One significant outcome from this work, evident from Figure 4 and Table 1, are the remarkable dielectric constants for Region III at long DT (>50 s) with the highest directly observed value 3.5 × 10^11^ (350 billion) for a discharge time of the 10 layer F-SDM of >200 s. Clearly, F-SDM are superdielectrics. One particular feature of the dielectric constants is that they are not constant, but rather increase with increasing thickness. It is revealing to compare this behavior with that of P-SDM for which the “dielectric constants” are constant with thickness [3,4,5], and with T-SDM [1,2], for which the dielectric constants were found to increase approximately as 1/t^2^, where t is thickness of the dielectric layer. The increasing values of “dielectric constant” for the F-SDM illustrate a behavior “between” that of the P-SDM and the T-SDM. That is, any increase is inconsistent with observed P-SDM behavior, but the measured rate of increase was much less than that found for T-SDM.

### 2.3. Energy Density

**Figure 5:**
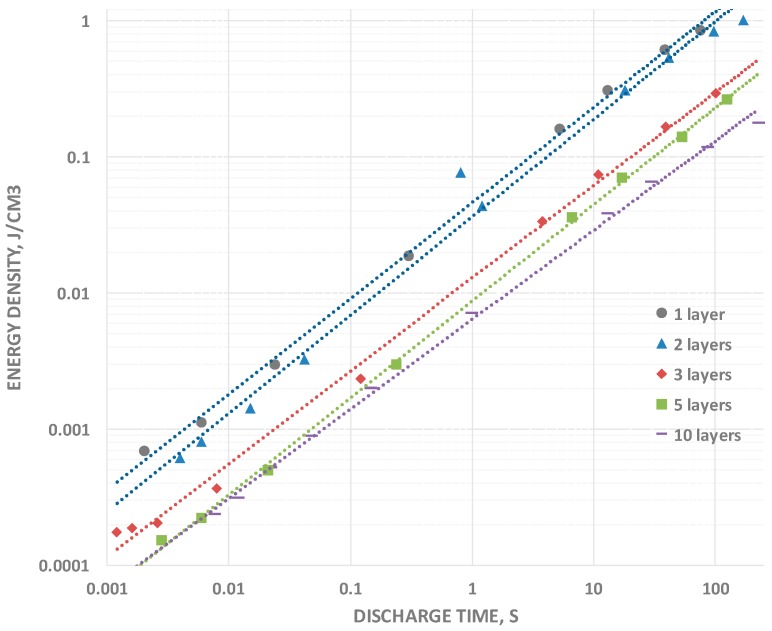
Energy Density vs. Discharge Time. The initial energy density at long discharge times is highest for one layer and decreases with each added layer, but the power law relationship (slope) between energy density and discharge time is very similar for all capacitors studied.

Energy density data were obtained in two steps. First, the area under the entire constant current curve, 2.3–0.1 V, is integrated to yield “volt-seconds”, this is multiplied by the constant current to yield energy (integration of “power”, where power is volts × current). This procedure is required because energy cannot be determined from the usual simple relationship between energy and capacitance because capacitance changes with voltage. Nonetheless, there is a smooth relationship between energy and discharge time. Energy, like capacitance, rolls off smoothly for all capacitors tested as the discharge times are reduced (Figure 5). Once again, the roll-off rates are nearly “parallel” independent of the number of layers. An interesting feature of the data is that, even for the relatively thick 1 layer capacitor (360 μ), an energy density greater than 1 J/cm^3^ was directly measured for very long discharge times. Although not even close to that recorded for T-SDM [1,2], relative to the best barium titanate based electrostatic capacitors this is a remarkable value [6,7,8].

The increasing energy density with decreasing thickness suggests that a thin fabric type SDM could rival the TSDM energy density observed in earlier studies. Indeed, a conservative extrapolation, based on a the empirical finding of one order of magnitude increase in net energy density for each one order of magnitude decrease in thickness (see Table 1), and an energy density at 100 s twice that at 50 s, indicates an energy density of >75 J/cm^3^ for a 5 micron thick nylon layer and discharge time of 100 s.

### 2.4. Power Density

**Figure 6:**
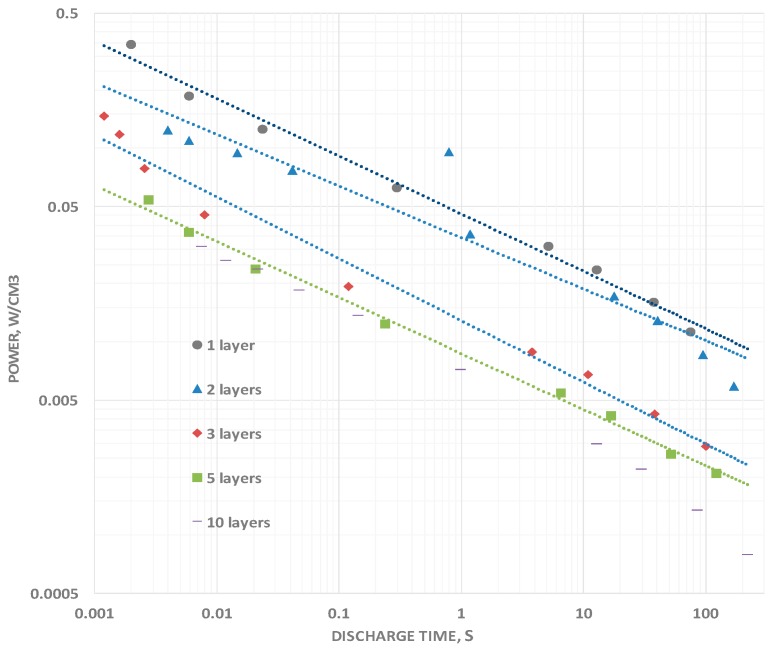
Power vs. Discharge Time. The power observed decreases monotonically with the number of layers. Notably, the power increases with decreasing discharge times. This is because the roll-off of energy is slower than the “roll-off” of discharge time.

The power delivery as a function of frequency clearly increases as the discharge times are reduced (Figure 6). Power was computed simply by determining the total energy, computed as described above, and dividing it by the discharge time. The increase in power delivered at a discharge time of 0.01 s, a typical high power application time, is more than an order of magnitude greater, in all cases, than the power delivered during a 100 s discharge. The measured increase in power with decreasing DT is anticipated for any system for which the capacitance roll off is less than 1 dB per 1 dB decrease in discharge time. That is, as long as the exponent in Equation (1) is greater than negative one, power will increase with decreasing DT value.

**Figure 7:**
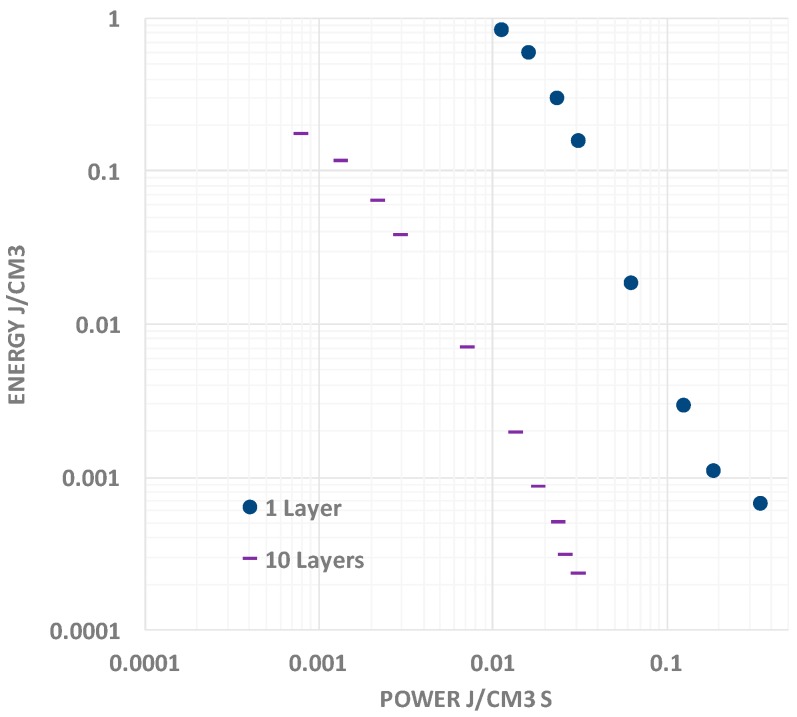
Ragone Plot Presentation. The data obtained for two of the F-SDM based capacitors studied are shown. As with other types of capacitors, as the energy density decreases, the power output increases. The basis for this relationship is that as the discharge time decreases, the energy density decreases, but more slowly. Thus, the energy delivered per unit time (power) increases with decreasing discharge time.

It should be noted that the typical “Ragone chart” (Figure 7) presentation of data reflects the same aspects of behavior seen in the above capacitance, energy and power vs. discharge time plots [9]. Specifically, as frequency increases (nearly equivalent to decreasing discharge time), the capacitance rolls off. Given the linear relationship between energy and capacitance, as frequency increases, energy density decreases. As shown in Figure 7, the Energy vs. Power curve can be readily fit with a single power law for F-SDM. In contrast, for many supercapacitors, apparently, there is a change in “power law” such that power output reaches a maximum even as energy continues to drop.

## 3. Discussion

The basis for the SDM hypothesis: Dielectrics increase capacitance by reducing the field everywhere relative to the no dielectric case. Specifically, any dielectric exposed to a field created by charge on electrodes forms dipoles that create fields “opposite” the field generated by the charge on the electrodes. Relative to the “dielectric free” situation, this reduces the net electric field strength everywhere. In turn, this reduces the work, that is the integral of electric field over any path (voltage), necessary to bring another charge to the electrode surface. Thus, when a dielectric is present between parallel plates, more charge is necessary to create the same voltage reached in the absence of a dielectric. This is equivalent to increasing the charge/voltage ratio, which is the definition of increasing capacitance. Hence, any dielectric that can produce dipoles larger than those in solids barium titanate should have a dielectric constant higher than that found for that material. The above understanding motivated the design of the present work. To wit: A nylon fabric saturated with “salt water” should have dipoles as long as the thickness of the nylon, at least thousands of times larger than that of any solid, including BaTiO_3_. Thus the dielectric constant of an SDM is predicted to be much higher than that of any solid.

**Figure 8:**
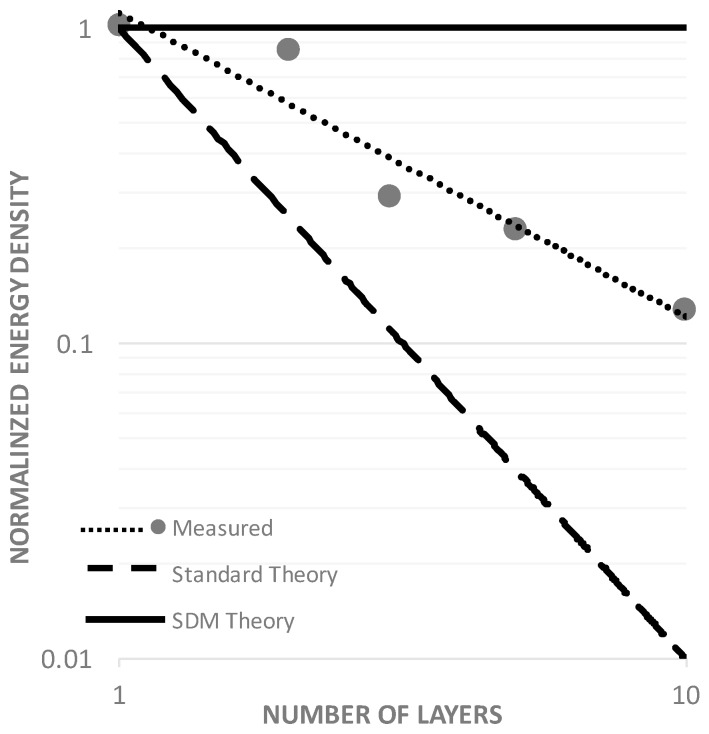
Energy Density vs. Thickness. According to SDM theory (**solid** line), the energy density is independent of thickness. According to standard theory (**dashed** line) the energy density falls as inverse thickness squared. The actual data (**points/dotted** line) for energy density as a function of dielectric thickness fall between these two theories.

The first major empirical finding supports the above predicted high dielectric: the measured dielectric constants are above 10^7^ (vs. <10^4^ for barium titanate) even at very short discharge times, establishing F-SDM as a new class of SDM. Second, the capacitance and dielectric constant changes with voltage in a very similar pattern to that for aqueous based P-SDM and T-SDM. That is, the capacitance changes with voltage, but can be well modeled as constant in three voltage zones for discharge times less than ca. 25 s. Third, the relatively slow increase in dielectric constant with increasing layer thickness demonstrates this class of materials is not like P-SDM, and, the measured and clear drop in energy density with dielectric layer thickness indicates behavior that is distinct from T-SDM. The behavior of F-SDM is unique (Figure 8). Fourth, there is a typical pattern decrease in capacitance, dielectric constant and energy density with decreasing discharge time. Fifth, extrapolating the energy density values for the relatively thick samples studied suggests that capacitors made with thin fabric layers (ca. 5 μ) might have exceptional energy densities (ca. >75 J/cm^3^).

A low surface area nylon mesh containing a salt solution is predicted to have a high dielectric constant by the SDM theory, but not by any other theory. A brief review shows no viable alternative models in the literature. Alternative theories of high dielectric constants include high dielectric constants at the percolation threshold [10,11,12], a nano metal particle model [13,14,15], and a quantum “surface state” model for colossal dielectric behavior [16,17,18,19,20]. As discussed elsewhere, none of these models appears to provide a reasonable framework for understanding the current system [1,2,3,4,5]. Specifically, there is no conceivable percolation process [21], there are no metal particles, and there are no surface states associated with the liquid phase. Finally, NPS are clearly not a variation on electric double layer capacitors (EDLC), as the NPS capacitors are missing two elements of standard supercapacitors: a high surface area conductive electrode (e.g., graphene), and a thin separator to allow ion transport, but to keep the two high surface area electrodes from touching/shorting [22,23].

Another issue requiring discussion is the “in-between” PSDM and TSDM behavior observed. In P-SDM dipole length, critical in determining dielectric constant, is established by the pore structure of the powder employed. Pore structure does not change with thickness, hence the dielectric constant is unchanging, as observed [3,4,5]. Energy density drops, consequently, as expected (thickness^−2^) for any standard dielectric material. In T-SDM, the dipole length is determined by the length of the open pores in the titania structure. These pores run the width of the oxide layer. Thus, as the dipole length increases exactly at the same rate as the dielectric layer thickness, the amount of salt also increases proportional to the pore length, and concomitantly so does the dielectric constant. This is not “standard” behavior for dielectric materials, but is anticipated for TSDM as explained in earlier publications [1,2]. Moreover, the theory of energy density for a system of ever increasing pore length is that energy density should be largely independent of dielectric thickness, as observed.

It is argued that pore length change with increasing dielectric layer thickness for an F-SDM is between those observed for PSDM and TSDM. The pore length is anticipated to increase with dielectric layer thickness, but at a slower rate than that of the dielectric layer itself. That is, it is anticipated that there is not perfect alignment between the layers of nylon fabric. Hence, liquid containing holes (~50% by area) in the mesh only partially “line up”, limiting the average pore length increase with the addition of each layer. This limits the average increase in dipole length with increasing number of layers. The average dipole length does increase with each layer added, but not as quickly as the overall thickness. This qualitative explanation is consistent with the observed change in energy density with number of layers.

The final point of interest is the decrease in dielectric constant and concomitantly energy density with decreases in discharge time. A reasoned extrapolation of the SDM theory suggests this is to be expected. Indeed, each time the applied field is reversed the original dielectric constant will not be recovered until all the ions in the solution within a pore physically move by diffusion, or field assisted convection, to the “other side” of the pore. This takes time. If the discharge time is too short, complete reversal of the ions will not take place, leading to a smaller net dipole strength within the pore, and hence a lower net dielectric constant. As the charge time gets shorter, the net motion is less, and hence the value of the dielectric constant should fall as the charge time decreases. This model leads to several questions for future work. First, will other dielectrics for which physical motion of ions is required show a similar fall off of capacitance as discharge time is reduced? In particular, will a similar discharge time dependence of dielectric constant be observed in EDLC? In addition, if the charging time is increased at any given current, will the energy density at any given discharge current increase?

## 4. Materials and Methods

Capacitor Fabrication: All capacitors, so-called Novel Paradigm Supercapacitors (NPS), were created from three components: nylon fabric, aqueous solutions of NaCl (30 wt %), and GTA grade Grafoil [24,25] electrodes (0.4 mm thick × 5 cm diameter), a commercially available, paper-like (~0.4 mm thick), moderate surface area (~20 m^2^/gm) material composed of compressed graphite (>99%). Nylon fabric squares, ~5.1 cm on a side, nominal thickness 0.36 mm, 50% open space, were dipped into the salt solution for approximately one hour, and then smoothed onto a Grafoil electrode. For the multi-layer samples, additional salt solution saturated fabric layers were added one at a time. The second Grafoil electrode was then placed on top and the thickness of the capacitor determined from an average of four measurements with a micrometer. Once the capacitor “sandwich” was created, in order to retard drying, it was placed on a small plastic block, and the block placed in a plastic bag containing water saturated cloth (3).

Measurement: The capacitive behavior was determined using a BioLogic Model SP 300 Galvanostat (Bio-Logic Science Instruments SAS, Claix, France) in constant current charge/discharge mode. Data were collected from the smaller of these intervals: 0.01 s or 0.01 V Other methods were judged to be inappropriate for determination of characteristics as prior SDM studies clearly show that the capacitance of NPS is a strong function of voltage. Hence, methods such as impedance spectroscopy that measure the dielectric constant at a single voltage, generally 0 ± 15 mV [8,9], do not yield the full story regarding energy storage characteristics. For a reliable understanding of the behavior expected for energy storage devices, the behavior over the entire voltage operating range must be studied [6,26,27].

At each selected constant current at least ten complete cycles were recorded, and generally twenty. This method does not permit the selection of the discharge rate; however, the discharge rate is a function of the discharge current. Thus, the trend in dielectric constants and energy density was determined by variation of the controllable parameter, discharge current. In all cases the charging current was the same magnitude as the discharge current, but of opposite sign.

There are several potential sources of error in determination of capacitance and dielectric constant. First, is the measured current. Observation clearly showed this to be within 3% of the nominal current in all cases. A second source is in measurement of the capacitor thickness. In all cases, that was determined to be no greater than ±0.03 mm, which, for all capacitors studied, was less than 10%. Variation in the slope of the discharge (dI/dV), determined by measurement of multiple discharges, was found to be no more than 5%, indicating precision. Consideration of all these errors suggests the values presented in the results section are ±15% of true values. It is notable in this regard that a 220 μF commercial superdielectric capacitor (Maxwell) was studied as a “control” in the same circuit, over the same voltage range. The measured capacitance at 0.03 Hz was 215 μF, and was constant over nearly the entire voltage range.

The voltage range in most cases was selected to be between 2.3 and 0.1 volts (Figure 1) as earlier studies showed this range to be compatible with aqueous solution based SDM [1,2,3,4,5]. For illustration purposes, one example of a charge/discharge cycle operating between −2.3 and +2.3 volts is shown (Figure 2).

## Figures and Tables

**Table 1:** Dielectric and Energy Density as a function of number of layers for 50 s DT.

Layers	Thickness (cm)	Approx. Dielectric Constant * DT ** 50 s	Approx. Energy Density (J/cm^3^) ^ DT ** 50 s
1	0.036	5 × 10^9^	0.7
2	0.074	2 × 10^10^	0.6
3	0.114	3 × 10^10^	0.2
5	0.191	5 × 10^10^	0.15
10	0.38	1.2 × 10^11^	0.08

* Dielectric constants listed are for the voltage range 0.8–0.1 volts. Over this voltage range, the dielectric constant was in fact constant. As noted in the text, the value of the dielectric constant decreases above ~0.8 V. ^ The energy density is not constant but decreases with increasing thickness. ** DT—Discharge time. Defined in text.
